# Important modifications by sugammadex, a modified γ-cyclodextrin, of ion currents in differentiated NSC-34 neuronal cells

**DOI:** 10.1186/s12868-016-0320-5

**Published:** 2017-01-03

**Authors:** Hung-Te Hsu, Yi-Ching Lo, Yan-Ming Huang, Yu-Ting Tseng, Sheng-Nan Wu

**Affiliations:** 1 Graduate Institute of Medicine, Kaohsiung Medical University, Kaohsiung City, 80708 Taiwan; 2 Department of Anesthesia, Kaohsiung Municipal Ta-Tung Hospital, Kaohsiung City, 80145 Taiwan; 3 Department of Pharmacology, School of Medicine, Kaohsiung Medical University, Kaohsiung City, 80708 Taiwan; 4 Department of Physiology, National Cheng Kung University Medical College, No. 1, University Road, Tainan City, 70101 Taiwan; 5 Graduate Institute of Natural Products, School of Pharmacy, Kaohsiung Medical University, Kaohsiung City, 80708 Taiwan

**Keywords:** Sugammadex, Motor neuron, Delayed-rectifier K^+^ current, Activation kinetics, L-type Ca^2+^ current, Glucocorticoid

## Abstract

**Background:**

Sugammadex (SGX) is a modified γ-cyclodextrin used for reversal of steroidal neuromuscular blocking agents during general anesthesia. Despite its application in clinical use, whether SGX treatment exerts any effects on membrane ion currents in neurons remains largely unclear. In this study, effects of SGX treatment on ion currents, particularly on delayed-rectifier K^+^ current [*I*_K(DR)_], were extensively investigated in differentiated NSC-34 neuronal cells.

**Results:**

After cells were exposed to SGX (30 μM), there was a reduction in the amplitude of *I*_K(DR)_ followed by an apparent slowing in current activation in response to membrane depolarization. The challenge of cells with SGX produced a depolarized shift by 15 mV in the activation curve of *I*_K(DR)_ accompanied by increased gating charge of this current. However, the inactivation curve of *I*_K(DR)_ remained unchanged following SGX treatment, as compared with that in untreated cells. According to a minimal reaction scheme, the lengthening of activation time constant of *I*_K(DR)_ caused by cell treatment with different SGX concentrations was quantitatively estimated with a dissociation constant of 17.5 μM, a value that is clinically achievable. Accumulative slowing in *I*_K(DR)_ activation elicited by repetitive stimuli was enhanced in SGX-treated cells. SGX treatment did not alter the amplitude of voltage-gated Na^+^ currents. In SGX-treated cells, dexamethasone (30 μM), a synthetic glucocorticoid, produced little or no effect on L-type Ca^2+^ currents, although it effectively suppressed the amplitude of this current in untreated cells.

**Conclusions:**

The treatment of SGX may influence the amplitude and gating of *I*_K(DR)_ and its actions could potentially contribute to functional activities of motor neurons if similar results were found in vivo.

**Electronic supplementary material:**

The online version of this article (doi:10.1186/s12868-016-0320-5) contains supplementary material, which is available to authorized users.

## Background

Sugammadex (SGX) is recognized as a modified γ-cyclodextrin with a lipophilic core and a hydrophilic periphery, and it has been used clinically for reversal of neuromuscular blockade caused by rocuronium or vecuronium during general anesthesia [1–5]. A previous report showed that addition of SGX could cause neuronal apoptosis in primary cultures [6]. This compound per se was also reported to be effective at reversing neurodegenerative disorder of the lower motor neurons [7, 8]. However, interestingly, how the treatment with SGX can perturb ionic currents remains largely unexplored.

The K_V_3 channels, a subfamily of K_V_ channels, are distinguished from other types of K_V_ channels by more positively shifted voltage-dependent activation and by faster activation and deactivation rates [9, 10]. These differences enable K_V_3 channels to be major determinants of high-frequency firing existing in several types of central neurons [11–15]. The activity of K_V_3.1 channels has been recently described as playing a crucial role in controlling the amplitude of action potentials at synapses [16]. The de novo mutations in *KCNC1*, which encodes K_V_3.1 channels, have been also found to expand phenotypic spectra of this channel to progressive myoclonus epilepsy [17]. Therefore, these channels clearly are important targets used for investigations on electrical behaviors of central neurons including motor neurons [10].

The NSC-34 mouse motor neuron cell line is a hybridoma cell line derived from the fusion of mouse neuroblastoma with motor neuron-enriched spinal cord cells [18, 19]. These cells may create an easily accessible and clonally uniform motor neuron-like cell line that overcomes the problems associated with the culturing of primary spinal motor neurons. It has been demonstrated to be a suitable model for investigations on the mechanisms of neuronal development and differentiation in vitro and for studying electrophysiological properties of motor neurons in spite of being not considered as an adult motor neuron [18–20]. The biophysical properties of delayed-rectifier K^+^ current [*I*_K(DR)_] in NSC-34 cells were previously found to resemble the K_V_3.1-encoded current because of positive mRNA detection of K_V_3.1 (*KCNC1*) [21, 22]. As NSC-34 cells were differentiated, the density of *I*_K(DR)_ was significantly enhanced. Previous work from our laboratory has shown that removal of membrane cholesterol by methyl-β-cyclodextrin, a cyclic oligosaccharide, could modify activation kinetics of *I*_K(DR)_ in motor neuron-like cells [23]. Additionally, although voltage-gated Na^+^ current (*I*_Na_) in NSC-34 neuronal cells has been previously reported [24], few studies have been concerned with the biophysical or pharmacological properties of Ca^2+^ currents in these cells.

Therefore, the purpose of this work was to test whether SGX treatment could exert any perturbations on ionic currents present in NSC-34 neuronal cells differentiated with low serum and retinoic acid. The biophysical and pharmacological properties of ionic currents including delayed-rectifier K^+^ current [*I*_K(DR)_], voltage-gated Na^+^ current (*I*_Na_) and L-type Ca^2+^ current (*I*_Ca,L_) in untreated and SGX-treated cells have been characterized and compared in this study. Interestingly, the present results indicate that SGX treatment is capable of modifying the activation kinetics of *I*_K(DR)_ elicited by membrane depolarization in these cells in a concentration-, time-, and state-dependent manner.

## Methods

### Drugs and solutions

Sugammadex (Bridion^®^, SGX, C_72_H_104_Na_8_O_48_S_8_) was obtained from Schering-Plough (Kenilworth, NJ, USA), aconitine, dexamethasone (DEX), l-aspartic acid, neostigmine, nifedipine, retinoic acid, tetraethylammonium chloride (TEA) and tetrodotoxin were from Sigma-Aldrich (St. Louis, MO, USA), iberiotoxin, apamin and ω-conotoxin GVIA were from Alomone Labs (Jerusalem, Israel), isobavachalcone (2′,4′,4-trihydroxy-3′-[3″-methylbut-3″-enyl]chalcone) was from Enzo Life Sci. (Plymouth Meeting, PA, USA), midazolam was from Nang Kuang Pharmaceutical Co. (Tainan City, Taiwan) and ranolazine [(±)-*N*-(2,6-dimethyl-phenyl)-4[2-hydroxy-3(2-methoxy-phenoxy)propyl]-1-piperazine acetamide] was from Tocris Cookson, Ltd. (Bristol, UK). For cell preparations, all culture media, fetal bovine serum (FBS), l-glutamine, trypsin/EDTA, penicillin–streptomycin and amphotericin B were obtained from Invitrogen (Carlsbad, CA, USA). All other chemicals including CdCl_2_, CsCl, CsOH and MgSO_4_ were obtained from regular commercial chemicals and of reagent grade. The compositions of bathing and pipette solutions used in this study are illustrated in Table 1.

**Table 1:** Composition of normal Tyrode’s solution and the pipette solution used in this study

Solution	Purpose or name	Composition
Bathing solution	Normal Tyrode’s solution	136.5 mM NaCl, 5.4 mM KCl, 1.8 mM CaCl_2_, 0.53 mM MgCl_2_, 5.5 mM glucose, and 5.5 mM HEPES–NaOH buffer, pH 7.4
Pipette solution	For recordings of K^+^ current or membrane potential	130 mM K-aspartate, 20 mM KCl, 1 mM KH_2_PO_4_, 1 mM MgCl_2_, 3 mM Na_2_ATP, 0.1 mM Na_2_GTP, 0.1 mM EGTA, and 5 mM HEPES–KOH buffer, pH 7.2
	For recordings of Na^+^ or Ca^2+^ currents	130 mM Cs-aspartate, 20 mM CsCl, 1 mM KH_2_PO_4_, 1 mM MgCl_2_, 3 mM Na_2_ATP, 0.1 mM Na_2_GTP, 0.1 mM EGTA, and 5 mM HEPES–CsOH buffer, pH 7.2

### Cell preparation and differentiation

NSC-34 neuronal cells were originally produced by fusion of the motor neuron-enriched, embryonic mouse spinal cords with the mouse neuroblastoma [19]. These cells were kindly provided by Professor Yuh-Jyh Jong (Department of Pediatrics, Kaohsiung Medical University Hospital, Kaohsiung City, Taiwan). They were routinely grown in 1:1 mixture of Dulbecco’s modified Eagle medium (DMEM) and Ham’s F12 medium that was supplemented with 10% (v/v) FBS and 1% penicillin–streptomycin. Cultures were incubated at 37 °C in a humidified environment of 5% CO_2_/95% air. The medium was replenished every 2–3 days for removal of non-adhering cells. To slow cell proliferation and enhance their maturation towards a differentiated state [20], before confluence, cells were grown in 1:1 DMEM plus Ham’s F12 medium supplemented with low serum (1% FBS) and 1 μM retinoic acid. The SGX-treated cells were incubated at 37 °C for 1 h in normal Tyrode’s solution containing different concentrations of this compound. The reason that the duration was set at 1 h was to ensure that SGX could exert its interaction with cell membrane.

### Electrophysiological measurements

Shortly before each experiment, cells were dissociated, and an aliquot of cell suspension was transferred to a homemade recording chamber positioned on the stage of a CKX-41 inverted microscope (Olympus, Tokyo, Japan). Cells were immersed at room temperature (20–25 °C) in normal Tyrode’s solution containing 1.8 mM CaCl_2_. The patch electrodes used were prepared from Kimax capillary tubes (#34500; Kimble Glass, Vineland, NJ, USA) using a vertical two-step electrode puller (PP-83 or PP-830; Narishige, Tokyo, Japan), and their tips were then fire-polished with an MF-83 micro-forge (Narishige). Experiments were performed using the whole-cell configuration of standard patch-clamp technique using either an RK-400 (Bio-Logic, Claix, France) or an Axopatch 200B (Molecular Devices, Sunnyvale, CA, USA) patch-clamp amplifier [19]. Junctional potentials that developed when the composition of the pipette solution was different from that in the bath were nulled.

### Data recordings

The signals consisting of voltage and current tracings were displayed and recorded online using an ASUSPRO-BU401LG computer (ASUS, Taipei City, Taiwan) equipped with a Digidata 1440A device (Molecular Devices), and the experiments were controlled by pCLAMP 10.2 software (Molecular Devices). Current signals were low-pass filtered at 3 kHz and digitized at 10 kHz. In some experiments of verifying analog-to-digital conversion, signals are digitized using a PowerLab acquisition system with LabChart 7.0 programs (AD Instruments, Gerin, Tainan City, Taiwan). The resultant data achieved during this experiment were analyzed off-line by use of various analytical tools including the LabChart 7.0 program (Gerin), Origin 8.0 (OriginLab, Northampton, MA, USA) and custom-made macro procedures run under Excel 2013 (Microsoft, Redmond, WA, USA). The voltage-step profiles digitally created from pCLAMP 10.2 were employed to evaluate current–voltage (*I*–*V*) relationships or steady-state inactivation of ionic currents [e.g., *I*_K(DR)_].

### Data analyses

The relationships between the relative *I*_K(DR)_ amplitude and the membrane potential obtained with or without the treatment of SGX (30 μM) were fitted with a Boltzmann function of the following form:

$$\frac{I}{{I_{ \hbox{max} } }} = \frac{1}{{1 + \exp \left[ {\frac{{ - (V - V_{1/2} )qF}}{RT}} \right]}},$$

where *I*_max_ is the maximal amplitude of *I*_K(DR)_ elicited by membrane depolarization, *V*_1/2_ the voltage at which there is half-maximal activation, *q* the apparent gating charge, *F* Faraday’s constant, *R* the universal gas constant, and *T* the absolute temperature.

The free energy involved in the gating of *I*_K(DR)_ (Δ*G*_0_) was calculated assuming that there is a 2-state (i.e., closed [resting] and open) gating model inherently in the channel. Δ*G*_0_ for *I*_K(DR)_ activation at 0 mV with or without treatment of SGX would be equal to *q* × *F* × *V*_1/2_ [25, 26]. The standard errors of Δ*G*_0_ (i.e., $\upsigma_{{{\text{qFV}}_{1/2} }}$) were calculated according to:

$$\sigma_{{qFV_{1/2} }} = F \times \sqrt {V_{1/2}^{2} \sigma_{q}^{2} + q^{2} \sigma_{{V_{1/2} }}^{2} }$$

where σ_q_ and $\upsigma_{{{\text{V}}_{1/2} }}$ represent the standard errors in *q* and *V*_1/2_ respectively.

Perturbation by SGX treatment of free energy involved in the gating of *I*_K(DR)_ was calculated as ΔΔ*G*_0_ = Δ*G*_0_^SGX^ − Δ*G*_0_^Ctrl^ = *F*(*qV*_1/2SGX_ − *qV*_1/2Ctrl_) = Δ(*qFV*_1/2_), where Δ*G*_0_^Ctrl^ and Δ*G*_0_^SGX^ indicate the free energy of *I*_K(DR)_ activation taken from untreated cells and cells exposed to SGX respectively.

The steady-state inactivation curve of *I*_K(DR)_ with or without treatment of SGX was derived and plotted against the conditioning pulses and then fitted to another Boltzmann equation:

$$\frac{I}{{I_{ \hbox{max} } }} = \frac{1}{{1 + \exp \left[ {\frac{{(V - V_{1/2} )}}{k}} \right]}},$$

where *V* represents the conditioning potential in mV, *V*_1/2_ is the membrane potential for half-maximal inactivation, and *k* is the slope factor of inactivation curve for *I*_K(DR)_ elicited by membrane depolarization.

Linear or nonlinear curve-fitting to data sets presented herein was performed using either Microsoft Solver function embedded in Excel (Microsoft) or Origin 8.0 program (OriginLab). The values are provided as the mean ± standard error of the mean (SEM) with sample sizes (*n*) indicating the cell number from which the results were obtained. The paired or unpaired Student’s *t* test and a one-way analysis of variance with the least-significant difference method for multiple comparisons were used for statistical evaluation of differences among means. Statistical analyses were performed using the Statistical Package for Social Science 20 (SPSS; IBM Corp., Armonk, New York). Statistical significance was determined at a *P* value of <0.05.

## Results

### Effect of SGX treatment on delayed-rectifier K^+^ currents [*I*_K(DR)_] in differentiated NSC-34 neuronal cells

**Fig. 1:**
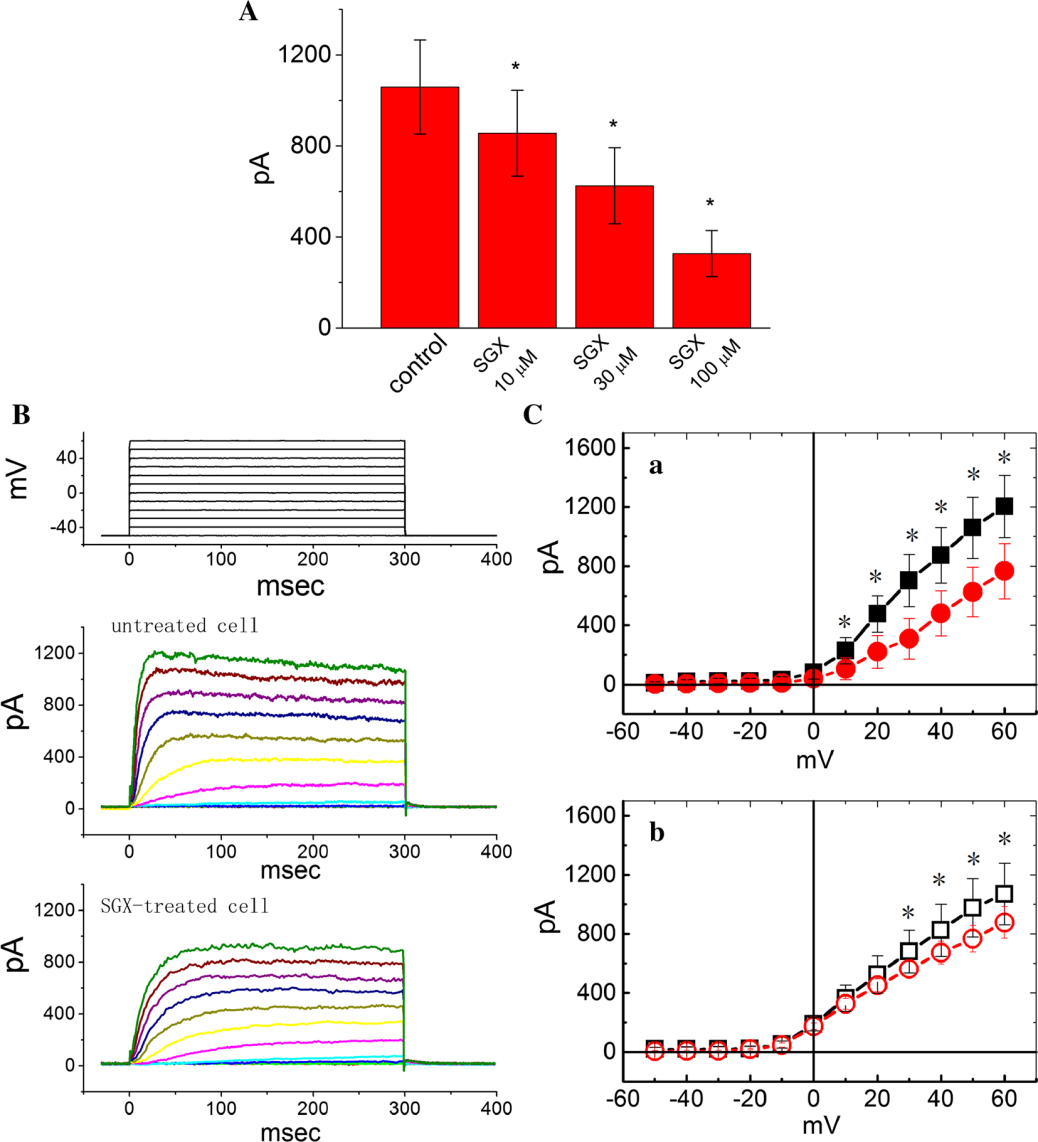
Effect of SGX treatment on *I*_K(DR)_ in differentiated NSC-34 neuronal cells. In these experiments, cells were bathed in Ca^2+^-free Tyrode’s solution containing 1 μM tetrodotoxin and the recording pipette was filled with K^+^-containing solution. The treated cells were incubated with SGX (30 μM) for 1 h at 37 °C. (**A**)* Bar graph* showing the data of *I*_K(DR)_ amplitude when cells were treated with 10, 30 and 100 μM SGX. Current amplitudes were measured at the beginning of depolarizing voltage was obtained at the 60th milliseconds after the initial rise of voltage from −50 to +50 mV (mean ± SEM; n = 10–12 for *each bar*). *Significantly different from control (*P* < 0.05). (**B**) Superimposed current traces obtained in untreated (*upper*) and SGX-treated (*lower*) cells. The cells examined were held at −50 mV and the voltages ranging from −50 to +60 mV in 10-mV increments were applied, as whole-cell recordings were established. The uppermost part indicates the voltage protocol used. (**C**) Current amplitude versus membrane potential relationships of *I*_K(DR)_ in untreated cells (*square symbols*) and in cells treated with 30 μM SGX (*circle symbols*). In **C**(*a*) and **C**(*b*), *I*_K(DR)_ amplitude was measured at the beginning (*filled symbols*) and end (*open symbols*) of depolarizing steps, respectively. *I*_K(DR)_ amplitudes measured at the beginning of depolarizing voltage were obtained at the 60th milliseconds after the initial rise of voltage. Each point represents the mean ± SEM (n = 10–12). *Significantly different from controls [i.e., *I*_K(DR)_ amplitude at the same level of voltage step] (*P* < 0.05)

In the first set of experiments, the whole-cell configuration of the patch-clamp technique was conducted to investigate effects of SGX on ionic currents in these cells. Cells were bathed in Ca^2+^-free Tyrode’s solution that contained tetrodotoxin (1 μM) and 0.5 mM CdCl_2_, and the recording pipette was filled with K^+^-containing solution described in “Methods”. Figure 1A depicts the *I*_K(DR)_ amplitudes when cells were exposed to different concentrations (10, 30 and 100 μM) of SGX. Because the SGX concentration at 30 μM was close to the IC_50_ value for inhibition of *I*_K(DR)_, most experiments shown below used such concentrations. As illustrated in Fig. 1B, C, as the cell was held at −50 mV and different voltage steps ranging from −50 to +60 mV in 10-mV increments were applied, a family of large outward currents was readily elicited. Current amplitudes were increased with greater depolarizations in an outward-rectifying manner with a reversal potential of −76 ± 2 mV (n = 12). These outward currents, which were insensitive to inhibition by either iberiotoxin (200 nM) or apamin (200 nM), have been referred to as *I*_K(DR)_, the biophysical properties of which resemble the K_V_3.1-encoded K^+^ currents [12, 22]. In particular, as the cells were exposed to SGX (30 μM), the amplitude and gating of *I*_K(DR)_ were altered throughout the entire range of voltage-clamp steps as compared with those taken from untreated cells. For example, when the voltage step from −50 to +50 mV was evoked, SGX (10 μM) treatment significantly decreased the amplitude of initial *I*_K(DR)_ (i.e., the 60th millisecond after the beginning of voltage step) by 41 ± 3% from 1059 ± 207 to 625 ± 167 pA (n = 11, *P* < 0.05). However, such treatment slightly but significantly decreased *I*_K(DR)_ amplitude at the end of depolarizing pulse by 22 ± 2% from 976 ± 197 to 766 ± 89 pA (n = 11, *P* < 0.05). No significant difference in cell capacitance between untreated (17.9 ± 1.8 pF, n = 13) and SGX-treated (18.1 ± 1.9 pF, n = 13, *P* > 0.05) cells could be clearly demonstrated. The averaged amplitude versus voltage relationships of *I*_K(DR)_ were measured and plotted in untreated and SGX-treated cells (Fig. 1C). The *I*–*V* relationships with or without SGX treatment were then obtained at the beginning [Fig. 1C(a)] and end [Fig. 1C(b)] of voltage pulses; therefore, SGX suppressed *I*_K(DR)_ amplitude in a concentration-dependent manner. Additionally, neostigmine (1 μM), a typical drug used for reversing the effect of rocuronium-induced neuromuscular blockade, had no effect on the amplitude of gating of *I*_K(DR)_ in these cells (Additional file 1: Fig. S1).

### The activation curve of I_K(DR)_ obtained with or without treatment of SGX

**Fig. 2:**
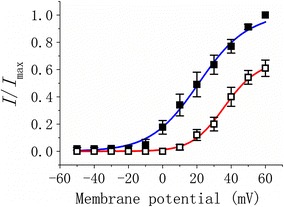
Effect of SGX on the activation curve of *I*_K(DR)_ recorded from differentiated NSC-34 neuronal cells. In these experiments, cells were bathed in Ca^2+^-free Tyrode’s solution. Voltage dependence of *I*_K(DR)_ activation in the absence (*filled square symbol*) and presence (*open square symbol*) of SGX (30 μM) treatment (mean ± SEM; n = 9 for each point) is illustrated. The smooth lines obtained in untreated and SGX-treated cells represent the best fits to the Boltzmann equation described in “Methods”. Notably, SGX treatment can shift the activation curve of *I*_K(DR)_ to a depolarizing voltage, together with increase in the gating charge of this current

Figure 2 shows the activation curve of *I*_K(DR)_ in the absence and presence of SGX (30 μM) treatment. The plot of relative *I*_K(DR)_ amplitude as a function of membrane potential was determined and fitted with a Boltzmann function as described under “Methods”. In untreated cells, *V*_1/2_ = 21.1 ± 2.2 mV and *q* = 1.93 ± 0.04 *e* (n = 9), while in SGX-treated cells, *V*_1/2_ = 36.1 ± 1.9 mV and *q* = 2.98 ± 0.17 *e* (n = 9). The data showed that, as differentiated NSC-34 cells were treated with SGX (30 μM), the activation curve of this current was shifted along the voltage axis to more positive potentials by approximately 15 mV and the elementary charge for activation was elevated 1.5-fold.

According to the values of *q* and *V*_1/2_, the free energy involved in the gating of *I*_K(DR)_ at 0 mV (Δ*G*_0_) in the absence and presence of SGX treatment was estimated to be 3.93 ± 0.12 and 10.35 ± 0.18 kJ/mol (n = 9) respectively. The perturbation by SGX treatment of free energy (ΔΔ*G*_0_) involved in *I*_K(DR)_ gating was calculated to be 6.42 ± 0.15 kJ/mol. It is therefore anticipated from these data that SGX treatment can increase the free energy needed for *I*_K(DR)_ activation observed in differentiated NSC-34 cells.

### Kinetic evaluation of I_K(DR)_ block by SGX

**Fig. 3:**
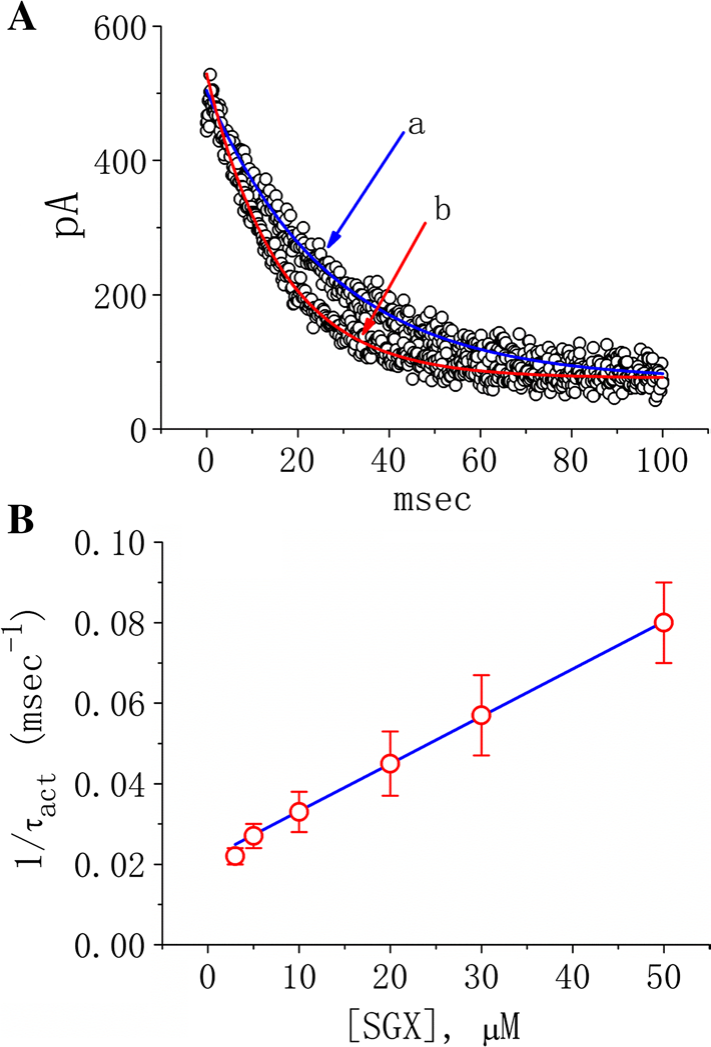
Evaluation of the kinetics of SGX-induced block of *I*_K(DR)_. The *I*_K(DR)_ established by depolarizing pulses from −50 to +50 mV was measured with cell treatment of different SGX concentrations. In **A**, activation time courses of SGX-sensitive *I*_K(DR)_ (τ_act_) obtained during cell exposure to 10 μM (*a*) and 30 μM (*b*) SGX were fitted by a single exponential with a value of 27.1 and 15.9 ms respectively. SGX-sensitive current was taken by subtracting current in SGX-treated cells from that in untreated ones. In **B**, the reciprocal of activation time constant (i.e., 1/τ_act_) of SGX-sensitive current was plotted against the SGX concentration. Data points shown in *open circles* were well fitted by a linear regression, indicating that there is a molecularity of one. According to reaction scheme, blocking (*k*_+1_) and unblocking (*k*_−1_) rate constants for SGX-induced blocks of *I*_K(DR)_ were calculated to be 0.0012 ms^−1^ μM^−1^ and 0.021 ms^−1^ respectively. Mean ± SEM (n = 8–11 for each point)

During cell exposure to SGX, the activation time course of *I*_K(DR)_ in response to membrane depolarization tended to become slower. The activation kinetics of SGX-induced block of *I*_K(DR)_ in response to membrane depolarization was further quantitatively evaluated in cells exposed to different SGX concentrations. The concentration dependence of *I*_K(DR)_ inhibition by SGX treatment is illustrated in Fig. 3. The results showed that its effects on *I*_K(DR)_ could exert a concentration-dependent increase in the rate of development of inhibition. Consequently, this effect on *I*_K(DR)_ in differentiated NSC-34 cells can be explained by a state-dependent blocking mechanism in which this compound may preferentially bind to the closed (resting) state of the K_V_ channels according to a minimal kinetic scheme:

$${\text{O}}\underset{\alpha }{\overset{\text{B}}{\longleftrightarrow}}{\text{C}}\underset{{k_{ - 1} }}{\overset{{k_{ + 1} \cdot \left[ B \right]}}{\longleftrightarrow}}{\text{C}} \cdot {\text{B}},$$

where α and β represent kinetic constants for the opening and closing of the K_V_ channel respectively; *k*_+1_ and *k*_−1_ are those for blocking and unblocking by SGX treatment; and [B] is the SGX concentration. C, O and C·B indicate the closed (resting), open, and closed-blocked state respectively.

Blocking and unblocking rate constants, *k*_+1_ and *k*_−1_, could be determined from activation time constants of SGX-sensitive *I*_K(DR)_ (i.e., difference in *I*_K(DR)_ taken from untreated and SGX-treated cells) obtained in different concentrations of this compound. The rate constants were then computed using the relation:

$$\frac{1}{{\tau_{act} }} = k_{ + 1} \left[ B \right] + k_{ - 1} ,$$

where *k*_+1_ and *k*_−1_ respectively resulted from the slope and from the y-axis intercept at [B] = 0 of the linear regression interpolating the reciprocal time constants (1/τ_act_) versus the SGX concentration. The relationship between 1/τ_act_ and [B] was computed to be linear with a correlation coefficient of 0.95 (Fig. 3B). The blocking and unblocking rate constants were thereafter calculated to be 0.0012 ms^−1^ μM^−1^ and 0.021 ms^−1^ respectively. According to these rate constants, dividing *k*_−1_ by *k*_+1_ yielded a dissociation constant (*K*_D_) of 17.5 μM under SGX treatment.

### Inability of SGX to modify the steady-state inactivation curve of I_K(DR)_

**Fig. 4:**
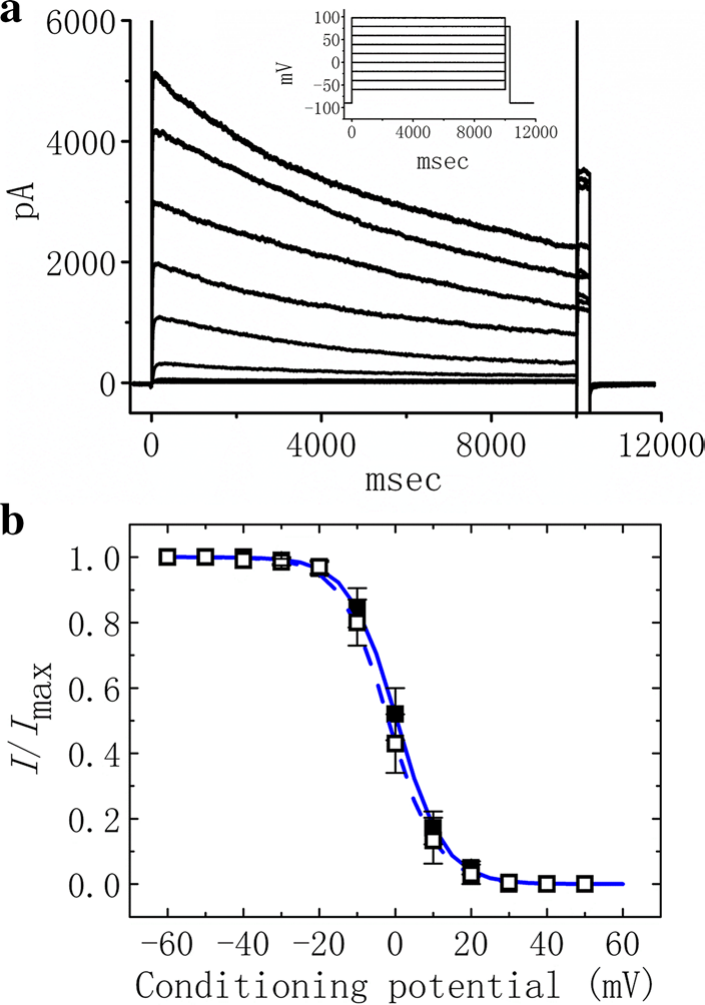
Steady-state inactivation of *I*_K(DR)_ obtained in the absence and presence of SGX treatment. With the aid of a two-step voltage protocol, the steady-state inactivation parameters of *I*_K(DR)_ in differentiated NSC-34 cells were evaluated. **a** Superimposed current traces obtained in an untreated cell. *Inset* in **a** indicates the voltage protocol used in this set of experiments. **b** Normalized amplitudes of *I*_K(DR)_ (*I*/*I*_max_) obtained in the control (*filled square symbol*) and during cell exposure to 30 μM SGX (*open square symbol*) were constructed against the conditioning potential. The smooth curves derived from untreated and SGX-treated cells were fitted by the Boltzmann equation as described in “Methods”. Each point represents the mean ± SEM (n = 9)

In order to further characterize effect of SGX treatment on *I*_K(DR)_, we next studied the inactivation of *I*_K(DR)_ in differentiated cells using a two-step voltage protocol. The quasi-inactivation curves of *I*_K(DR)_ in untreated and SGX-treated cells are illustrated in Fig. 4. In this set of experiments, a 10-s conditioning pulse in different membrane potentials preceded a test potential (300 ms in duration) to +80 mV from a holding potential of −90 mV (Fig. 4a). The relationships between the conditioning pulses and the normalized amplitudes of *I*_K(DR)_ in untreated and SGX-treated cells were constructed and fitted by the Boltzmann equation described in “Methods”. In untreated cells, *V*_1/2_ = 6.2 ± 0.9 mV and *k* = 0.42 ± 0.02 mV (n = 9), while in SGX-treated cells, *V*_1/2_ = 6.3 ± 0.09 mV and *k* = 0.44 ± 0.03 mV (n = 9). Distinguishable from the results of *I*_K(DR)_ activation curve obtained in untreated and SGX-treated cells, the values for both *V*_1/2_ and *k* were not found to differ significantly between the two groups of cells (*P* > 0.05). The experimental results therefore suggest that there is little or no modification in the voltage-dependent profile of *I*_K(DR)_ inactivation following SGX treatment.

### The increase of cumulative inhibition of I_K(DR)_ activation in SGX-treated cells

**Fig. 5:**
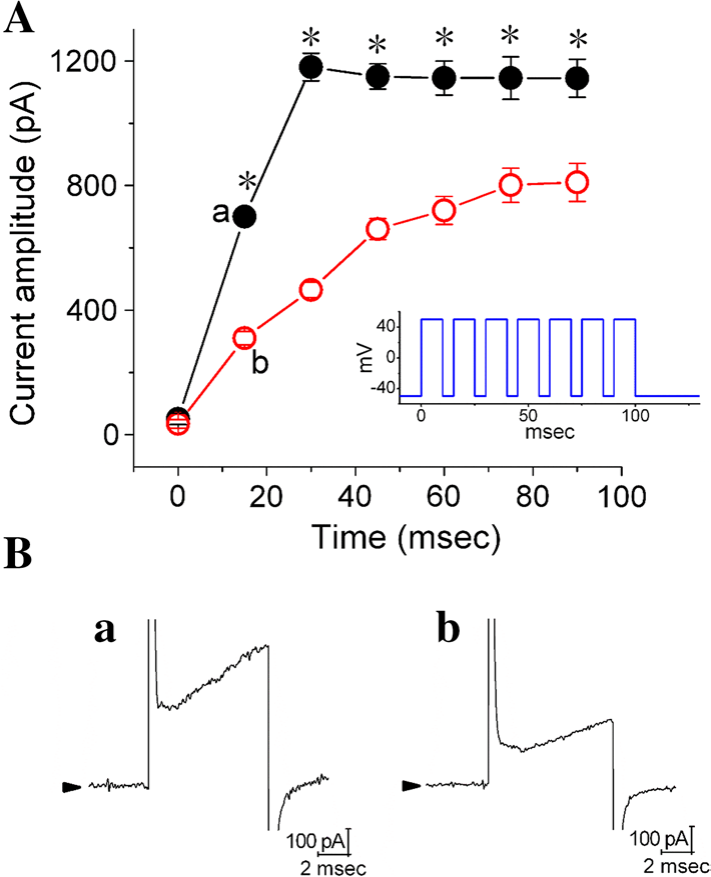
Time course of repetitive cumulative activation of *I*_K(DR)_ obtained with or without SGX treatment. In **A**, the peak amplitude of each *I*_K(DR)_ was measured during repetitive depolarizations from −50 to +50 mV with a duration of 10 ms at a rate of 70 Hz. *Filled* and *open circles* were obtained in untreated and SGX-treated cells respectively. *Inset* indicates the voltage protocol used. In **B**, original *I*_K(DR)_ traces (*a* and *b*) in response to rapid membrane depolarization from −50 to +50 mV were shown. Traces *a* and *b* correspond to the data points labeled *a* and *b* in **A**. *Arrowhead* in traces *a* and *b* indicate the zero current level. Notably, in addition to inhibition of *I*_K(DR)_ amplitude, SGX treatment can slow the rate of excessive cumulative activation of *I*_K(DR)_ in response to repetitive stimuli. *Significantly different from the data from untreated cells (*P* < 0.05). Mean ± SEM (n = 9–10)

In another set of experiments, we sought to determine whether in SGX-treated cells, the activation time course of *I*_K(DR)_ induced by repetitive depolarizing stimuli could be altered. Under control conditions (i.e., in untreated cells), a single 100-ms depolarizing step to +50 mV from a holding potential of −50 mV was applied to produce an exponential increase of *I*_K(DR)_ with a time constant of 27 ± 2 ms (n = 10). However, the activation time constant for 10-ms repetitive pulses to +50 mV, each of which lasted 10 ms with 5-ms interval at −50 mV between the depolarizing pulses, was significantly reduced to 19 ± 2 ms (n = 10, *P* < 0.05). The results indicate a progressive increase in the activation rate of *I*_K(DR)_ in response to repetitive depolarizing stimuli (Fig. 5 and Additional file 2: Fig. S2). However, in SGX-treated cells, the value of activation time constant obtained during this train of short repetitive pulses became raised. In the cells exposed to 30 μM SGX, the time constants were significantly increased to 47 ± 5 ms (n = 9, *P* < 0.05); accordingly, the results showed that there was an excessive accumulative slowing in the activation of *I*_K(DR)_ as cells were exposed to SGX.

### Effect of SGX treatment on voltage-gated Na^+^ current (I_Na_) in differentiated NSC-34 cells

**Fig. 6:**
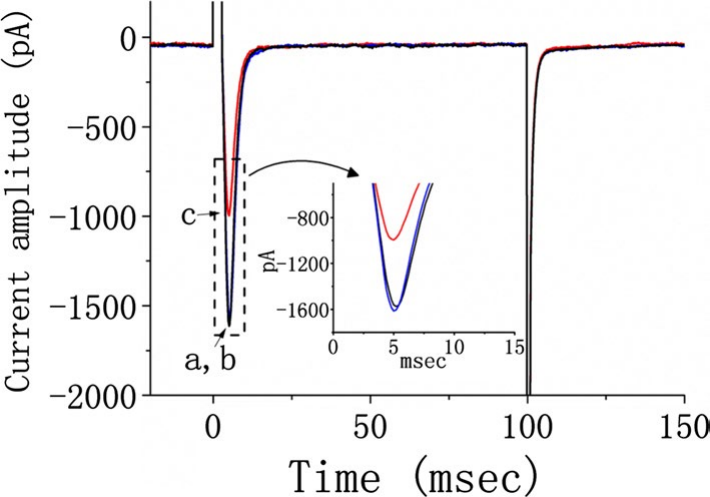
Effect of SGX treatment on voltage-gated Na^+^ current (*I*_Na_) in differentiated NSC-34 cells. The untreated and SGX-treated cells were bathed in Ca^2+^-free Tyrode’s solution. *I*_Na_ was evoked in response to membrane depolarization from −80 to −10 mV. *a* Superimposed *I*_Na_ tracings obtained in the cell without (*a*) and with (*b*) the exposure to SGX (30 μM). *I*_Na_ trace labeled (*c*) was obtained after addition of ranolazine (10 μM) in SGX-treated cells. The *inset* indicates an expanded record from *dashed box*. Notably, *I*_Na_ amplitude obtained from untreated and SGX-treated cells did not differ significantly; however, ranolazine could effectively suppress *I*_Na_ recorded from SGX-treated cells

In the next set of experiments, we investigated effect of SGX treatment on *I*_Na_ in these cells. Untreated or SGX-treated cells were bathed in Ca^2+^-free Tyrode’s solution containing 10 mM TEA and the recording pipette was filled with Cs^+^-containing solution, the composition of which is shown in Table 1. The *I*_Na_ was elicited by depolarizing pulse from −80 to −10 mV with a duration of 100 ms. In our experiments, SGX treatment at 10, 30, and 100 μM did not exert any significant effect on the peak amplitude of *I*_Na_ or *I*_Ca,L_ in these cells. As shown in Fig. 6, there was no significant difference in the amplitude of *I*_Na_ between untreated cells (1.82 ± 0.3 nA, n = 8) and cells treated with SGX (10 μM) (1.81 ± 0.2 nA, n = 8, *P* > 0.05). However, in SGX-treated cells, addition of ranolazine (10 μM), a blocker of *I*_Na_ [27], was capable of suppressing the peak amplitude of *I*_Na_ significantly from 1.82 ± 0.2 nA to 0.98 ± 0.1 nA (n = 7, *P* < 0.05). Therefore, SGX treatment per se did not modify the amplitude of *I*_Na_ in these cells, while the presence of ranolazine remained effective at suppressing *I*_Na_ in SGX-treated cells.

### Comparison of the effect of dexamethasone (DEX) on L-type Ca^2+^ current (I_Ca,L_) in SGX-treated cells

**Fig. 7:**
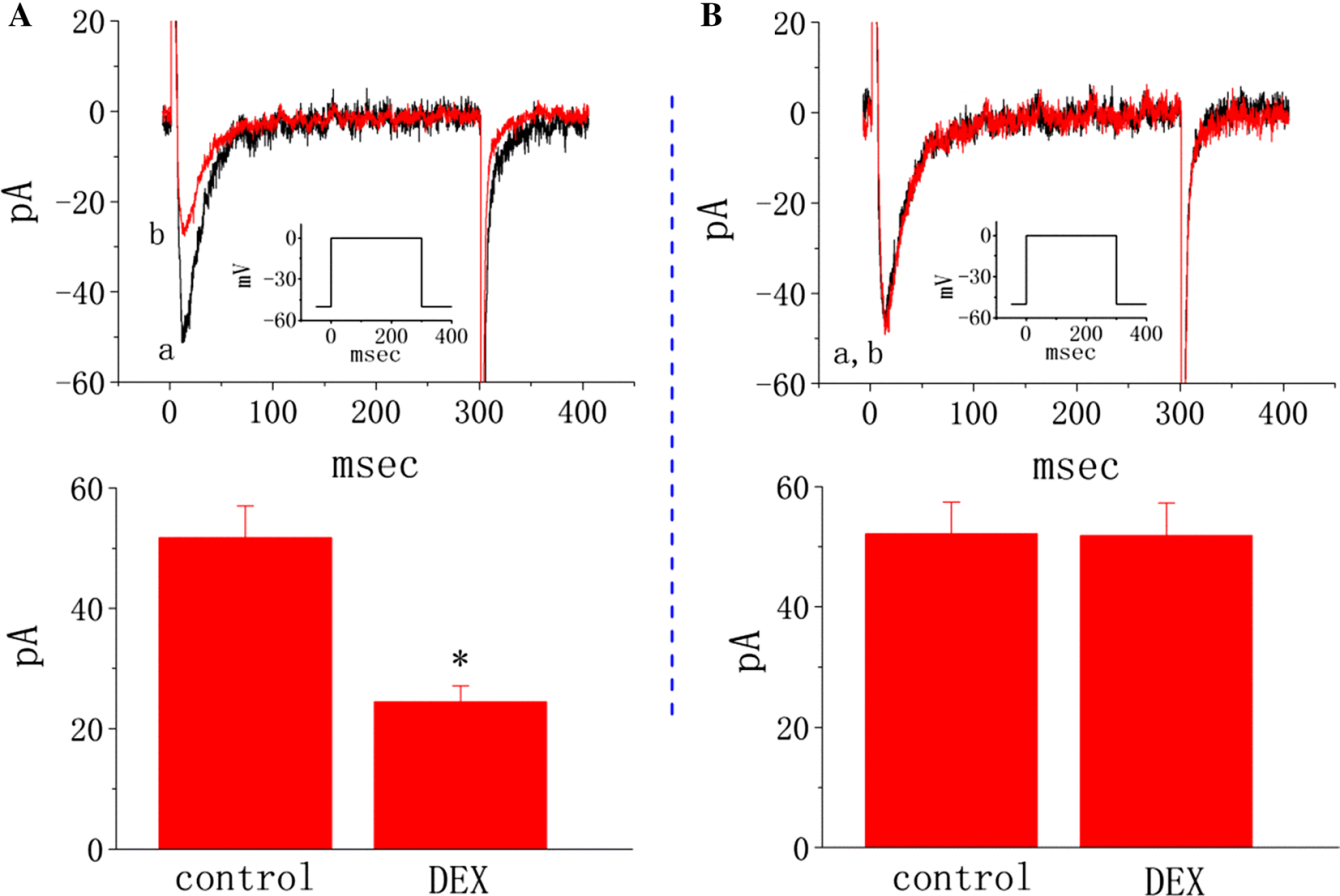
Effect of DEX on L-type Ca^2+^ current (*I*_Ca,L_) in untreated and SGX-treated cells. In these experiments, cells were bathed in normal Tyrode’s solution containing 1.8 mM CaCl_2_ and the recording pipette was filled with Cs^+^-containing solution. In the upper parts of **a** and **b**, superimposed *I*_Ca,L_ traces were obtained in untreated cells respectively. *I*_Ca,L_ traces labeled *a* in each panel are controls (i.e., in the absence of DEX) and those labeled *b* were obtained after addition of 30 μM DEX. *Inset* in the upper part of each panel indicates the voltage protocol used.* Bar graphs* shown in each panel indicate the summary of data showing inhibitory effect of DEX on the peak amplitude of *I*_Ca,L_ in untreated (**A**) and SGX-treated (**B**) cells (mean ± SEM; n = 11 for each bar). *Significantly different from control (*P* < 0.05). DEX: 30 μM dexamethasone

SGX is a modified γ-cyclodextrin and able to encapsulate steroid-like compounds [1, 4]. DEX, a synthetic glucocorticoid, can produce an inhibitory effect on the reversal of rocuronium-induced neuromuscular block by SGX in functionally innervated human muscle cells [28]. Therefore, in a final set of experiments, it was further examined whether the amplitude of *I*_Ca,L_ recorded from SGX-treated cells could be altered by DEX. The experiments were performed as cells were bathed in normal Tyrode’s solution containing 1.8 mM CaCl_2_ and 1 μM tetrodotoxin, and the recording pipette was filled with Cs^+^-containing solution. The peak amplitude of *I*_Ca,L_ in response to membrane depolarization from −50 to 0 mV was effectively suppressed by the presence of nifedipine (1 μM), a blocker of *I*_Ca,L_, but not by ω-conotoxin GVIA (1 μM), a toxin isolated from *Conus geographus* and known to block N-type Ca^2+^ current (Additional file 3: Fig. S3). As illustrated in Fig. 7, as untreated cells were depolarized from −50 to 0 mV, DEX (30 μM) significantly suppressed the peak amplitude of *I*_Ca,L_ from 51.7 ± 5.3 to 24.5 ± 2.6 pA (n = 11, *P* < 0.05); however, the overall *I*–*V* relationship of this current remained unchanged in the presence of DEX. The concentration of DEX (30 μM) used in this study was fundamentally based on a previous report [29]. The results are compatible with previous observations made in pituitary tumor cells [29]. In contrast, in SGX-treated cells, DEX at the same concentration had no significant effect on the amplitude of *I*_Ca,L_ [52.1 ± 5.3 pA (control), n = 11 versus 51.9 ± 5.4 pA (in the presence of DEX), n = 11, *P* > 0.05]; therefore, findings from these results indicate that, in SGX-treated cells, DEX-mediated inhibition of *I*_Ca,L_ in response to membrane depolarization was abolished.

## Discussion

In this study, the blocking of *I*_K(DR)_ by SGX treatment was noted to be not instantaneous, but to develop with time immediately after the cell became depolarized. Such treatment produces a time-dependent increase in the activation time constant of *I*_K(DR)_ in response to membrane depolarization. However, the inactivation curve of *I*_K(DR)_ obtained in untreated and SGX-treated cells did not differ significantly. It thus appears that, as cells are exposed to SGX, this compound has a greater affinity for the closed (resting) state in the K_V_ channel existing on differentiated NSC-34 cells. The activated channels during SGX treatment tended to produce a lower affinity than those residing in the closed state. As a result, the transition from closed to open state became slowed during cell exposure to SGX. Based on our study, it is therefore tempting to speculate that the treatment of SGX or other structurally similar agents binds to the closed state of the channel and/or blocks a prolonged channel closing in K_V_3.1 channels. Alternatively, the challenge of cells with SGX or other structurally similar agents can modulate K_V_3.1 channels where the closed channel conformation represents the high-affinity binding site. It also needs to be noted that, according to minimal reaction scheme shown here, the dissociation constant for SGX treatment was calculated to be 17.5 μM, a value that can be clinically achievable [1, 3, 5].

The K_V_3.1-encoded currents were reported to be the major molecular components of *I*_K(DR)_ in these cells [20, 21]. Importantly, our study demonstrated that the activation kinetics of *I*_K(DR)_ (i.e., K_V_3.1-encoded current) in SGX-treated cells virtually became slowed in a time- and state-dependent manner. The results were in contrast with inhibitory effects of midazolam or aconitine on *I*_K(DR)_ [12, 21]. These two compounds exerted inhibitory effects via a mechanism through binding to the open state of the channel followed by increased rate of *I*_K(DR)_ inactivation. During repetitive stimuli, the perturbation by SGX treatment of *I*_K(DR)_ activation was potentiated.

By virtue of computational analysis, previous work has shown that changes in activation kinetics of *I*_K(DR)_ might lead to generation of action potentials with spike-frequency adaptation [23]. The ability of K_V_3.1-encoded currents to control the waveforms of action potentials at synapses has recently been reported [16]. Indeed, different de novo mutations in *KCNC1* have been reported to display a wide variety of progressive myoclonus epilepsy [17]. However, SGX treatment had little or no effect on the peak amplitude of *I*_Na_. Therefore, the present results showing any changes in the amplitude and gating by SGX treatment of *I*_K(DR)_ can be of pharmacological and clinical relevance.

Following SGX treatment, *I*_K(DR)_ enriched in differentiated NSC-34 cells became activated at more depolarized voltages in comparison with that from untreated cells. Moreover, the steepness of activation curve for *I*_K(DR)_ became significantly greater in cells exposed to SGX, indicating that the effective number of elementary charges during channel activation in SGX-treated cells was significantly raised. These results are important because they led us to estimate that energy change (ΔG_0_^SGX^) for generation of *I*_K(DR)_ was a value of 10.35 kJ/mol. This value was found to be significantly greater than that ΔG_0_^Ctrl^ (i.e., 3.93 kJ/mol) in untreated cells. SGX treatment apparently is involved in voltage-sensitive gating functions of *I*_K(DR)_, despite no clear change in inactivation curve of *I*_K(DR)_ between the two groups of cells. The results lead us to propose that following SGX treatment, the energy barrier for activation of K_V_3.1 channels became elevated.

In our experimental conditions, supplementation of the medium with retinoic acid resulted in changes in cell morphology and an increase in mRNA expression of the K_V_3.1 subunit in differentiated NSC-34 neuronal cells [20, 21]. However, the modification of *I*_K(DR)_ kinetics by SGX presented here did not appear to occur by the gene regulation of these channels, because significant changes in this current in differentiated NSC-34 cells generally occurred with a short time course. Moreover, no changes in *I*_K(DR)_ density after treatment with SGX were observed, suggesting that such maneuver did not alter the main parts of ion channel permeation pathway (i.e., the S5 and S6 regions). It is thus possible that SGX treatment can regulate the gating kinetics of *I*_K(DR)_ with no apparent change in the number of functional channels on plasma membrane.

Consistent with previous studies [29], we clearly demonstrated that addition of DEX suppressed the peak amplitude of *I*_Ca,L_ in differentiated NSC-34 neuronal cells. It is important to note, however, that the inhibition by DEX of *I*_Ca,L_ did not occur in SGX-treated cells, despite the ability of MgSO_4_ to suppress *I*_Ca,L_ amplitude in both untreated and SGX-treated cells (data not shown). Previous observations have shown that DEX did not increase the activity of large-conductance Ca^2+^-activated K^+^ (BK_Ca_) channels in pituitary cells treated with methyl-β-cyclodextrin [30], suggesting that the binding of DEX to the protein(s) of BK_Ca_ channels relies on membrane cholesterol.

Whether DEX produces any significant effect on *I*_Na_ or *I*_K(DR)_ in NSC-34 cells or primary motor neurons needs to be further investigated. Whether the presence of SGX alters the cholesterol content in surface membranes and influences the DEX effect on *I*_Ca,L_ also remains to be explored. Nonetheless, our experimental results are consistent with earlier work showing that DEX is effective at exerting inhibitory effects on SGX reversal of rocuronium-induced neuromuscular block [28]. Alternatively, removal by SGX of DEX-induced block of *I*_Ca,L_ could be due mostly to the possibility that, similar to a mechanism by which it can reverse muscle relaxing effects by rocuronium, the SGX molecule can effectively encapsulate the DEX molecule [1, 3–5].

A recent report showed that methyl-β-cyclodextrin, a cholesterol-depleting agent, could induce activation of matrix metalloproteinase-2 (MMP-2) [31]. However, the reduction by SGX treatment of *I*_K(DR)_ activation rate observed in differentiated NSC-34 cells was unable to be reversed by isobavachalcone (10 μM) known to be an inhibitor of MMP-2 activity [32]. Therefore, alterations by SGX treatment of activation kinetics of *I*_K(DR)_ observed in differentiated NSC-34 cells are not closely associated with a mechanism linked to MMP-2 activation.

It should be noted that the pipette solution used in this study contained 3 mM ATP, which can adequately suppress the activity of ATP-sensitive K^+^ (K_ATP_) channels. The activity of K_ATP_ channels did not differ between the untreated and SGX-treated cells (data not shown). Changes by SGX treatment of *I*_K(DR)_ amplitude and gating observed in differentiated NSC-34 cells is unlikely to arise from inhibition of K_ATP_ channels.

The observed block of *I*_K(DR)_ caused by SGX treatment actually provides an intriguing mechanism for its inhibition that relies on the closed (resting) state of the K_V_3.1-encoded channels. The K_V_3.1-encoded currents were enriched in many central neurons including hippocampal pyramidal neurons, auditory neurons, and Purkinje cells [9, 11, 13, 14]. The activity of these K_V_ channels is recognized as participating in electrical behaviors of fast-spiking neurons [9, 10, 15, 16]. Challenging cells with SGX reduced the amplitude of *I*_K(DR)_ and slowed the activation time course of this current recorded from differentiated NSC-34 cells as well. The present observations would clearly initiate further studies to understand the SGX effects on electrical activity of motor neurons. Whether SGX-induced reversal of rocuronium-induced neuromuscular blockade is due partly to its blocking of *I*_K(DR)_ in motor neurons in vivo remains to be further investigated. Some adverse effect such as movement of a limb or the body may be partly explained by its inhibitory effect on *I*_K(DR)_.

It is noted that neostigmine, an inhibitor of acetylcholinesterase activity, is a typical drug used in anesthesia for reversing the effect of rocuronium-induced neuromuscular blockade. In our study, neostigmine (1 μM) did not exert any effect on the amplitude and gating of *I*_K(DR)_ in differentiated NSC-34 cells (Additional file 1: Fig. S1). Findings from our study might explain previous observations showing that SGX could reverse more rapidly rocuronium-induced neuromuscular blockade [33] or that the treatment with SGX was associated with less frequent dry mouth than that of neostigmine [34]. Therefore, it remains to be further delineated whether SGX might exert differential actions when it is used with patients who have been administrated with DEX or other glucocorticoids [4], if similar findings presented here occur in vivo. Nonetheless, as motor neurons are exposed to SGX, the amplitude and gating of *I*_K(DR)_ could be modified and these actions might significantly contribute to functional activities of motor neurons.

## Conclusion

The SGX treatment may influence the amplitude and gating of *I*_K(DR)_ and its actions could contribute to functional activities of motor neurons if similar findings occurred in vivo.

## Additional files

**Additional file 1.** Lack of effect of neostigmine on *I*_K(DR)_ recorded from differentiated NSC-34 neuronal cells. In these experiments, cells were bathed in Ca^2+^-free Tyrode’s solution containing 1 μM tetrodotoxin and 0.5 mM CdCl_2_, and the recording pipette was filled with K^+^-containing solution. (A) Original current trace obtained in the absence (blue) and presence (red) of 1 μM neostigmine. Inset indicates the voltage protocol used. (B) Bar graph showing no significant effect of neostigmine (1 μM) on *I*_K(DR)_ amplitude measured at the end of depolarizing pulses (mean ± SEM; n = 8 for each bar; *P* > 0.05).

**Additional file 2.** Time course of *I*_K(DR)_ elicited by 100-ms depolarizing pulse from −50 to +50 mV (A) or repetitive cumulative activation (B) which was taken from the same NSC-34 cell. The number of raw data (indicated in filled circles) was reduced for clarity. Inset in (A) indicates the voltage protocol used. In (B), each *I*_K(DR)_ was evoked by 10-ms repetitive pulses to +50 mV, each of which lasted 10 ms with 5-ms interval. The activation time constants taken from (A) and (B) (indicated in smooth gray lines) are 25.4 and 19.5 ms, respectively.

**Additional file 3.** Effect of nifedpine and ω-conotoxin GVIA on *I*_Ca,L_ in differentiated NSC-34 neuronal cells. In these experiments, cells were bathed in normal Tyrode’s solution containing 1 μM tetrodotoxin and the recording pipette was filled with Cs^+^-containing solution. (A) Original *I*_Ca,L_ trace obtained in the absence (blue) and presence (red) of 1 μM nifedipine. Inset indicates the voltage protocol used. (B) Bar graph showing effect of nifedpine (1 μM) and ω-conotoxin GVIA (1 μM) on the peak amplitude of *I*_Ca,L_ in these cells (n = 7; mean ± SEM for each bar). * Significantly different from control (*P* < 0.05).

## Abbreviations

SGX: sugammadex
*I*_K(DR)_: delayed-rectifier K^+^ current
*I*_Na_: voltage-gated Na^+^ current
*I*_Ca,L_: L-type Ca^2+^ current
DEX: dexamethasone
TEA: tetraethylammonium chloride
FBS: fetal bovine serum
DMEM: Dulbecco’s modified Eagle medium
*I–V*: current versus voltage
Δ*G*_0_: the free energy involved in the gating of *I*_K(DR)_
SEM: standard error of the mean
ΔΔ*G*_0_: the perturbation by SGX treatment of free energy
BK_Ca_: large-conductance Ca^2+^-activated K^+^
MMP-2: matrix metalloproteinase-2
K_ATP_ channel: ATP-sensitive K^+^ channel
