# Supplementary material for: Important modifications by sugammadex, a modified γ-cyclodextrin, of ion currents in differentiated NSC-34 neuronal cells
Source: BMC Neurosci. 2017 Jan 3;18:6. doi: 10.1186/s12868-016-0320-5 (PMC5210182; doi:10.1186/s12868-016-0320-5)
Supplement: Supplementary file 1 — Additional file 1. Lack of effect of neostigmine on I K(DR) recorded from differentiated NSC-34 neuronal cells. In these experiments, cells were bathed in Ca2+-free Tyrode’s solution containing 1 μM tetrodotoxin and 0.5 mM CdCl2, and the recording pipette was filled with K+-containing solution. (A) Original current trace obtained in the absence (blue) and presence (red) of 1 μM neostigmine. Inset indicates the voltage protocol used. (B) Bar graph showing no significant effect of neostigmine (1 μM) on I K(DR) amplitude measured at the end of depolarizing pulses (mean ± SEM; n = 8 for each bar; P > 0.05). [file 12868_2016_320_MOESM1_ESM.docx]

**Figure S1.**








**Supplementary Figure 1.** Lack of effect of neostigmine on *I*_K(DR)_ recorded from differentiated NSC-34 neuronal cells. In these experiments, cells were bathed in Ca^2+^-free Tyrode’s solution containing 1 μM tetrodotoxin and 0.5 mM CdCl_2_, and the recording pipette was filled with K^+^-containing solution. (A) Original current trace obtained in the absence (blue) and presence (red) of 1 μM neostigmine. Inset indicates the voltage protocol used. (B) Bar graph showing no significant effect of neostigmine (1 μM) on *I*_K(DR)_ amplitude measured at the end of depolarizing pulses (mean±SEM; n=8 for each bar; *P*>0.05).
