# Supplementary material for: Important modifications by sugammadex, a modified γ-cyclodextrin, of ion currents in differentiated NSC-34 neuronal cells
Source: BMC Neurosci. 2017 Jan 3;18:6. doi: 10.1186/s12868-016-0320-5 (PMC5210182; doi:10.1186/s12868-016-0320-5)
Supplement: Supplementary file 2 — Additional file 2. Time course of I K(DR) elicited by 100-ms depolarizing pulse from −50 to +50 mV (A) or repetitive cumulative activation (B) which was taken from the same NSC-34 cell. The number of raw data (indicated in filled circles) was reduced for clarity. Inset in (A) indicates the voltage protocol used. In (B), each I K(DR) was evoked by 10-ms repetitive pulses to +50 mV, each of which lasted 10 ms with 5-ms interval. The activation time constants taken from (A) and (B) (indicated in smooth gray lines) are 25.4 and 19.5 ms, respectively. [file 12868_2016_320_MOESM2_ESM.docx]

**Figure S2.**








**Supplementary Figure 2.** Time course of *I*_K(DR)_ elicited by 100-msec depolarizing pulse from -50 to +50 mV (A) or repetitive cumulative activation (B) which was taken from the same NSC-34 cell. The number of raw data (indicated in filled circles) was reduced for clarity. Inset in (A) indicates the voltage protocol used. In (B), each *I*_K(DR)_ was evoked by 10-msec repetitive pulses to +50 mV, each of which lasted 10 msec with 5-msec interval. The activation time constants taken from (A) and (B) (indicated in smooth gray lines) are 25.4 and 19.5 msec, respectively.
