# Supplementary material for: Important modifications by sugammadex, a modified γ-cyclodextrin, of ion currents in differentiated NSC-34 neuronal cells
Source: BMC Neurosci. 2017 Jan 3;18:6. doi: 10.1186/s12868-016-0320-5 (PMC5210182; doi:10.1186/s12868-016-0320-5)
Supplement: Supplementary file 3 — Additional file 3. Effect of nifedpine and ω-conotoxin GVIA on I Ca,L in differentiated NSC-34 neuronal cells. In these experiments, cells were bathed in normal Tyrode’s solution containing 1 μM tetrodotoxin and the recording pipette was filled with Cs+-containing solution. (A) Original I Ca,L trace obtained in the absence (blue) and presence (red) of 1 μM nifedipine. Inset indicates the voltage protocol used. (B) Bar graph showing effect of nifedpine (1 μM) and ω-conotoxin GVIA (1 μM) on the peak amplitude of I Ca,L in these cells (n = 7; mean ± SEM for each bar). * Significantly different from control (P < 0.05). [file 12868_2016_320_MOESM3_ESM.docx]

**Figure S3.**








**Supplementary Figure 3.** Effect of nifedpine and ω-conotoxin GVIA on *I*_Ca,L_ in differentiated NSC-34 neuronal cells. In these experiments, cells were bathed in normal Tyrode’s solution containing 1 μM tetrodotoxin and the recording pipette was filled with Cs^+^-containing solution. (A) Original *I*_Ca,L_ trace obtained in the absence (blue) and presence (red) of 1 μM nifedipine. Inset indicates the voltage protocol used. (B) Bar graph showing effect of nifedpine (1 μM) and ω-conotoxin GVIA (1 μM) on the peak amplitude of *I*_Ca,L_ in these cells (n=7; mean±SEM for each bar). ^*^Significantly different from control (*P*<0.05).
